# Application of 3-D Microfluidic Models for Studying Mass Transport Properties of the Tumor Interstitial Matrix

**DOI:** 10.3389/fbioe.2019.00006

**Published:** 2019-01-23

**Authors:** Alex Avendano, Marcos Cortes-Medina, Jonathan W. Song

**Affiliations:** ^1^Department of Biomedical Engineering, The Ohio State University, Columbus, OH, United States; ^2^Department of Mechanical and Aerospace Engineering, The Ohio State University, Columbus, OH, United States; ^3^The Comprehensive Cancer Center, The Ohio State University, Columbus, OH, United States

**Keywords:** tumor engineering, microfabrication, extracellular matrix, cellular microenvironment, therapeutic testing

## Abstract

The physical remodeling associated with cancer progression results in barriers to mass transport in the tumor interstitial space. This hindrance ultimately affects the distribution of macromolecules that govern cell fate and potency of cancer therapies. Therefore, knowing how specific extracellular matrix (ECM) and cellular components regulate transport in the tumor interstitium could lead to matrix normalizing strategies that improve patient outcome. Studies over the past decades have provided quantitative insights into interstitial transport in tumors by characterizing two governing parameters: (1) molecular diffusivity and (2) hydraulic conductivity. However, many of the conventional techniques used to measure these parameters are limited due to their inability to experimentally manipulate the physical and cellular environments of tumors. Here, we examine the application and future opportunities of microfluidic systems for identifying the physiochemical mediators of mass transport in the tumor ECM. Further advancement and adoption of microfluidic systems to quantify tumor transport parameters has potential to bridge basic science with translational research for advancing personalized medicine in oncology.

## Introduction

Cancer has traditionally been described in terms of its molecular and genetic underpinnings, cellular heterogeneity, and network of signaling interactions during malignant progression (Hanahan and Weinberg, 2011). However, cancer can also be defined by its physicochemical features that arise due to function-altering mutations to the cellular constituents of the tumor microenvironment. For example, many solid tumor types, such as breast, pancreas, and liver, exhibit a desmoplastic response where stromal cells become hyperactivated leading to excessive extracellular matrix (ECM) production, growth of dense fibrotic tissue around the tumor (Trimboli et al., 2009; Kalluri, 2016), and subsequent tumor-promoting increases in mechanical stiffness (Leight et al., 2016; Reid et al., 2017). In addition to mechanical alterations, the tumor ECM may impede the distribution of macromolecules involved in regulating cell function (Netti et al., 2000). This hindrance to transport can also affect the ability of therapeutics to efficiently reach cancer cells. Consequently, the tumor ECM itself has emerged as a therapeutic target where the use of proteolytic enzymes and anti-fibrotic agents have been shown to normalize the ECM, improve drug penetration into the tumor, and increase patient survival (Nakai et al., 2012; Provenzano et al., 2012; Venning et al., 2015; Doherty et al., 2017; Papageorgis et al., 2017; Elahi-Gedwillo et al., 2018).

These newfound clinical implications of targeting the tumor ECM have heightened the importance of precise quantitative analysis of the mass transport properties of the tumor interstitial matrix. For this application, 3-D microfluidic systems, i.e., ones that integrate microscale technologies with 3-D tissue scaffolds, may provide a powerful approach because they possess the following desirable attributes : (1) bottom-up construction that enables tuning of ECM properties and spatial patterning of cellular constituents, (2) micron or cellular length scales where diffusive transport is critical, (3) controlled application of convective flow, and (4) typically favorable optical properties for real-time observation (Huh et al., 2011; Infanger et al., 2013; Akbari et al., 2017).

Here we highlight the application of microfluidic technologies for studying the transport properties of the tumor interstitium. To provide a balanced perspective, first we present a fundamental understanding of the physiological barriers to interstitial mass transport in tumors. We then discuss the parameters used to quantify transport in tumors and the established techniques used to measure these parameters. Lastly, we present prospective directions for the use of microfluidics as a tool for drug screening and development of targeted therapies. We note that in addition to the interstitium, other constituents of tumors such as the vasculature and transcellular membranes pose barriers to drug transport before reaching the intracellular space (Jain, 1987). For the foundational understanding of these other barriers in the context of tumors, we wish to direct the readers to these excellent review articles (Jain, 1988; Szakács et al., 2006; Dewhirst and Secomb, 2017).

## Fundamentals of Mass Transport in the Tumor Interstitium

### Physiological Barriers to Transport

**Figure 1:**
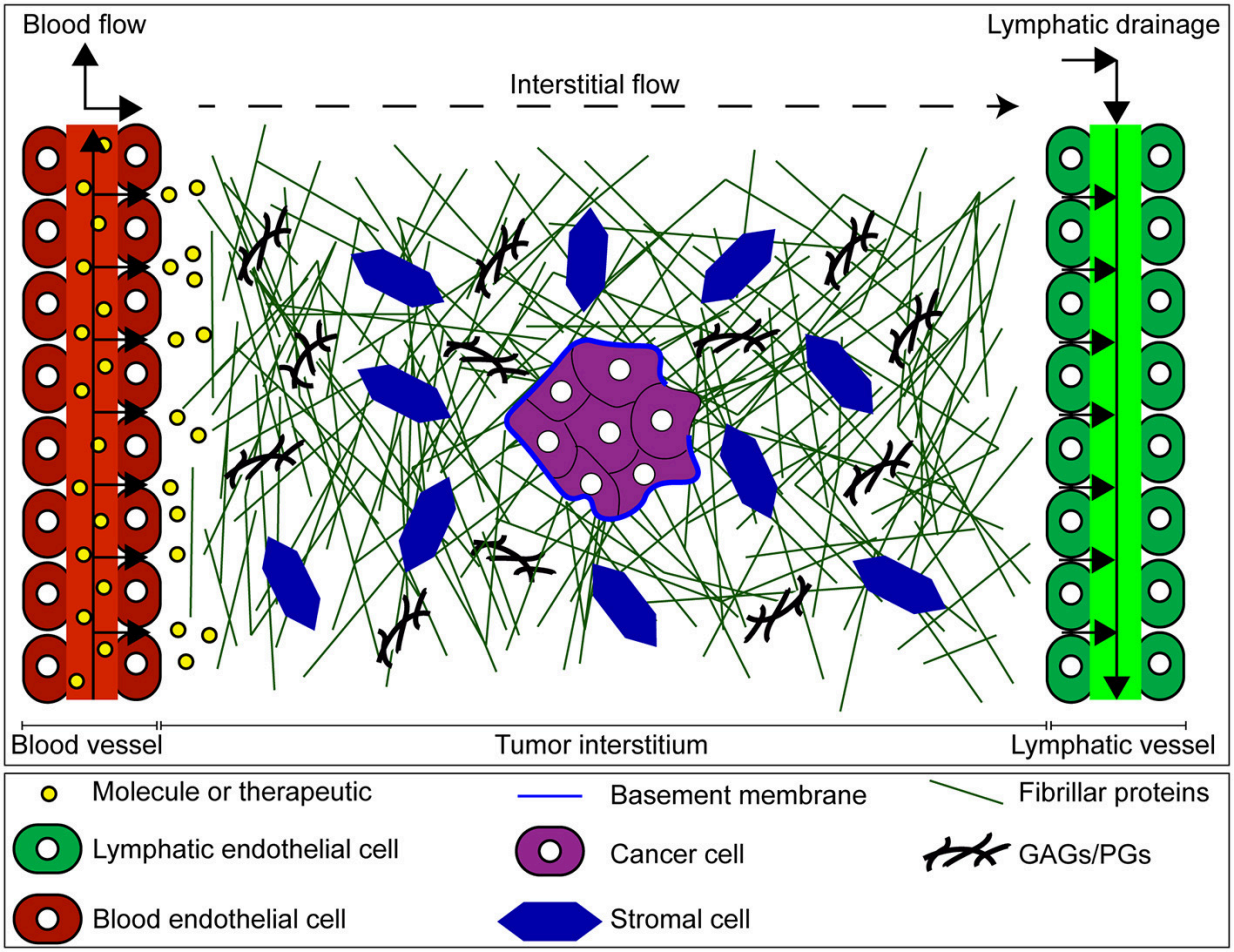
Mass transport through the tumor interstitium. Once extravasated from the vascular space, molecules must cross the tumor interstitium, and eventually drain through the lymphatic vessels. The tumor interstitium is occupied by the interstitial matrix composed of fibrillar and non-fibrillar components such as collagen, glycosaminoglycans (GAGs), proteoglycans (PGs), and basement membrane produced by both cancer and stromal cells. This matrix imposes barriers to transport of molecules in tumors, contributing to a more hostile malignancy.

The interstitial compartment (or interstitium) is comprised of an ECM, interstitial fluid, basement membrane proteins (e.g., collagen IV, laminin, elastin), and stromal cells (Figure 1). The interstitial ECM consists of a network of fibrous matrix proteins (e.g., collagen type I, fibronectin), glycosaminoglycans (GAGs) (e.g., hyaluronan, chondroitin sulfate), and proteoglycans (PGs) (e.g., versican, aggrecan, perlecan) (Wiig and Swartz, 2012; Theocharis et al., 2016; Xiong and Xu, 2016; Malandrino et al., 2018). Interstitial fluid is composed primarily of extravasated blood plasma and provides a medium for the transport of nutrients, waste products, and signaling molecules between cells of the interstitium.

The tumor interstitium poses unique barriers to transport which in turn can influence the delivery and uptake of therapeutic agents (Stylianopoulos et al., 2018). For example, the dense ECM of tumors hinders molecular diffusion (Jain, 1987; Pluen et al., 2001; Stylianopoulos et al., 2010; Zhang et al., 2017). In addition, tumors exhibit elevated interstitial fluid pressure (IFP) (Heldin et al., 2004; Elahi-Gedwillo et al., 2018) which can be attributed to excessive plasma leakage out of tumor blood vessels and impaired lymphatic function due to compressed vessels by growth-induced solid stress (Stylianopoulos et al., 2012; Nia et al., 2016). Uniformly high IFP in tumors limits interstitial convection as it abrogates the pressure difference between blood and lymphatic vessels. Also, fluid leakage from the tumor into surrounding tissue can result in outward radial flow that prevents transport of molecules into the tumor interior (Jain and Stylianopoulos, 2010). However, an important consideration is that these characteristics of tumors are highly heterogeneous, both within the same tumor and when comparing separate tumors (Jain and Stylianopoulos, 2010).

Transport through the tumor interstitial space relies on a combination of diffusion and convection (Jain, 1987; Netti et al., 1997; Kuszyk et al., 2001; Swartz and Fleury, 2007; Fan et al., 2014). The relative contributions of diffusive and convective transport can be quantified through the dimensionless parameter, the Peclet number (Pe):

(1) $$\begin{array}{l} Pe=\;\frac{convection}{diffusion}\;=\;\frac{Lv_{interstitial}}{D} \end{array}$$

Where *L* is the characteristic length associated with the transport of the molecule, *v*_*interstitial*_ is the interstitial fluid velocity, and *D* is the solute effective diffusion coefficient. In the interstitium, a typical *L* is ~100 μm (approximate distance between microvessels) (Dewhirst and Secomb, 2017). A typical *v*_*interstitial*_ is ~1 μm/s (Wiig and Swartz, 2012), although this value can be much lower or higher depending on the region of the tumor (Kingsmore et al., 2018). Finally, a representative value for *D* is ~10^−7^ cm^2^/s (Chary and Jain, 1989) (reported for serum albumin in both normal and neoplastic tissues). Further considerations for diffusion and convective transport in the tumor interstitium are discussed below.

### Diffusive Transport in the Tumor Interstitium

Molecular diffusion through the tumor interstitium is due to concentration gradients (Baish et al., 2011). Diffusive flux can be related to the concentration gradient through the effective diffusion coefficient *D* (cm^2^/s). In the case of one dimensional transport, this relationship is given by Ficks' Law:

(2) $$\begin{array}{l} J_{diff}=\;-D\frac{\delta \text{C}}{\delta \text{x}} \end{array}$$

where $\frac{\text{δC}}{\text{δx}}$ denotes the concentration gradient. The effective diffusion coefficient in the interstitium (or interstitial diffusivity) is determined by properties of both the molecule of interest and the interstitial matrix (Jain and Stylianopoulos, 2010). Properties of the molecule that affect its interstitial diffusivity include size, charge, and configuration (Jain, 1999; Jain and Stylianopoulos, 2010; Wiig and Swartz, 2012). The properties of the ECM in the tumor interstitium that affect the diffusivity of molecules include viscoelasticity, geometrical arrangement (e.g., collagen fiber orientation), and electrostatic properties (Swartz and Fleury, 2007; Seo et al., 2014). These properties are a consequence of ECM composition (e.g., collagen, GAG, and PG content) with collagen being the major determinant of interstitial diffusion (Netti et al., 2000).

### Convective Transport in the Tumor Interstitium

Convective transport of molecules through the tumor interstitium is driven by pressure gradients. The convective flux can be written as:

(3) $$\begin{array}{l} J_{conv}=v_{interstitial}fC \end{array}$$

where *C* is the concentration of the particle/molecule, *f* is the retardation coefficient (ratio of particle to fluid velocity) which is often assumed to equal 1, and *v*_*interstitial*_ is the interstitial fluid velocity. *v*_*interstitial*_ can be determined by the solution to the Brinkman equation for flow through a porous medium (Equation 4):

(4) $$\begin{array}{l} \mu \nabla^{2}v_{interstitial}-\frac{1}{K^{{\;}^{\prime }}}v_{interstitial}-\nabla p=0 \end{array}$$

where μ is the fluid viscosity, *K*′ is the hydraulic conductivity, and ∇*p* is the pressure gradient across the interstitium. Due to the large magnitude of surface drag relative to viscous dissipation, the viscous term in the Brinkman equation can often be neglected resulting in the more familiar Darcy's law. Darcy's law can then be used to write the convective flux in terms of the pressure gradient and *K*′:

(5) $$\begin{array}{l} J_{conv}=-CfK^{\prime }\frac{\delta P}{\delta x} \end{array}$$

where $\frac{\delta P}{\delta x}$ is the pressure gradient over a distance *x*. Thus, for a given $\frac{\delta P}{\delta x}$ the *K*′ of the interstitial ECM is the major determinant of interstitial velocity. *K*′ can also be expressed in terms of the Darcy permeability (also denoted as specific permeability) $K=\frac{K^{\prime }}{\mu }$ where μ is the viscosity of the fluid. This parameter is mostly dependent on the properties of the interstitial ECM including composition, geometrical arrangement, charge, and hydration (Levick, 1987; Ng and Swartz, 2003; Ng et al., 2005; Wiig and Swartz, 2012). Compared to normal tissue, tumors typically exhibit reduced *K*′ (Provenzano et al., 2012; Polydorou et al., 2014; Mpekris et al., 2017; Papageorgis et al., 2017).

## Measuring the Interstitial Transport Parameters in Tumors

Looking at the equations above for diffusive and convective fluxes, one can see that *D* and *K*′ primarily determine the effectiveness of these mode of transport within tumors. A brief overview on how these two parameters have been experimentally measured is discussed below.

### Interstitial Diffusivity (*D*)

Most techniques employed to measure *D* rely on measuring solute flux at a known concentration gradient or measuring relaxation of these gradients and fitting the diffusion equation to the data (Jain, 1999). Diffusion measurements *in vitro* have been performed using tissue slices or various gel or solution models of the interstitium (Jain, 1987; Pluen et al., 1999; Ramanujan et al., 2002). It should be noted that *D* is often lower than the diffusivity in free solution, and correlations have been developed that can relate both types (Swartz and Fleury, 2007). For *in vivo* settings, the use of intravital microscopy and fluorescence recovery after photobleaching (FRAP) has allowed the measurement of *D* (Chary and Jain, 2007). FRAP involves the use of a laser beam to artificially introduce a concentration gradient of a fluorescent tracer in a region of tissue and the relaxation of this gradient is analyzed to yield the diffusion coefficient and the convective velocity (Ramanujan et al., 2002).

### Hydraulic Conductivity (*K*′)

Measurements of *K*′ using Darcy's Law (Equation 5) involve estimating the flow rate under a known pressure gradient. This measurement is performed *in vitro* by applying flow across a tissue slice using an Ussing-style chamber (Hedbys and Mishima, 1962) or by ultracentrifugal sedimentation (Laurent and Pietruszkiewicz, 1961; Preston et al., 1965; Ethier, 1986). While measuring *K*′*in vivo* is comparatively more challenging, it has been achieved with the use of a micropore chamber (Swabb et al., 1974) as well as tail injections in rats (Swartz et al., 1999). *K*′ can also be estimated by confined compression testing of excised tumor chunks *ex vivo* (Polydorou et al., 2014; Mpekris et al., 2015; Papageorgis et al., 2017).

## Application of Microfluidic Models for Studying Tumor ECM Transport Properties

### Representing the Tumor ECM in Microfluidic Devices

While the techniques described above have collectively provided the framework for our understanding of transport in tumors, they have several limitations that are worthy of consideration. First, it is often very difficult to apply controlled perturbations *in vivo*. With regards to quantifying transport within tumors, it is immensely challenging to independently specify concentration and pressure gradients and subsequently decouple the contributions of diffusion and convection. Second, intravital microscopy used for FRAP experiments requires specialized equipment and training that may not be readily available to most laboratories. This imaging modality is also limited to only thin tissues or for the superficial layer of thick tissues. Third, an overarching challenge with the analysis of tumor tissue, whether assessed *in situ* or *ex vivo*, is the high degree of morphological heterogeneity present in tumors that may lead to uncertainties when interpreting experimental results.

**Figure 2:**
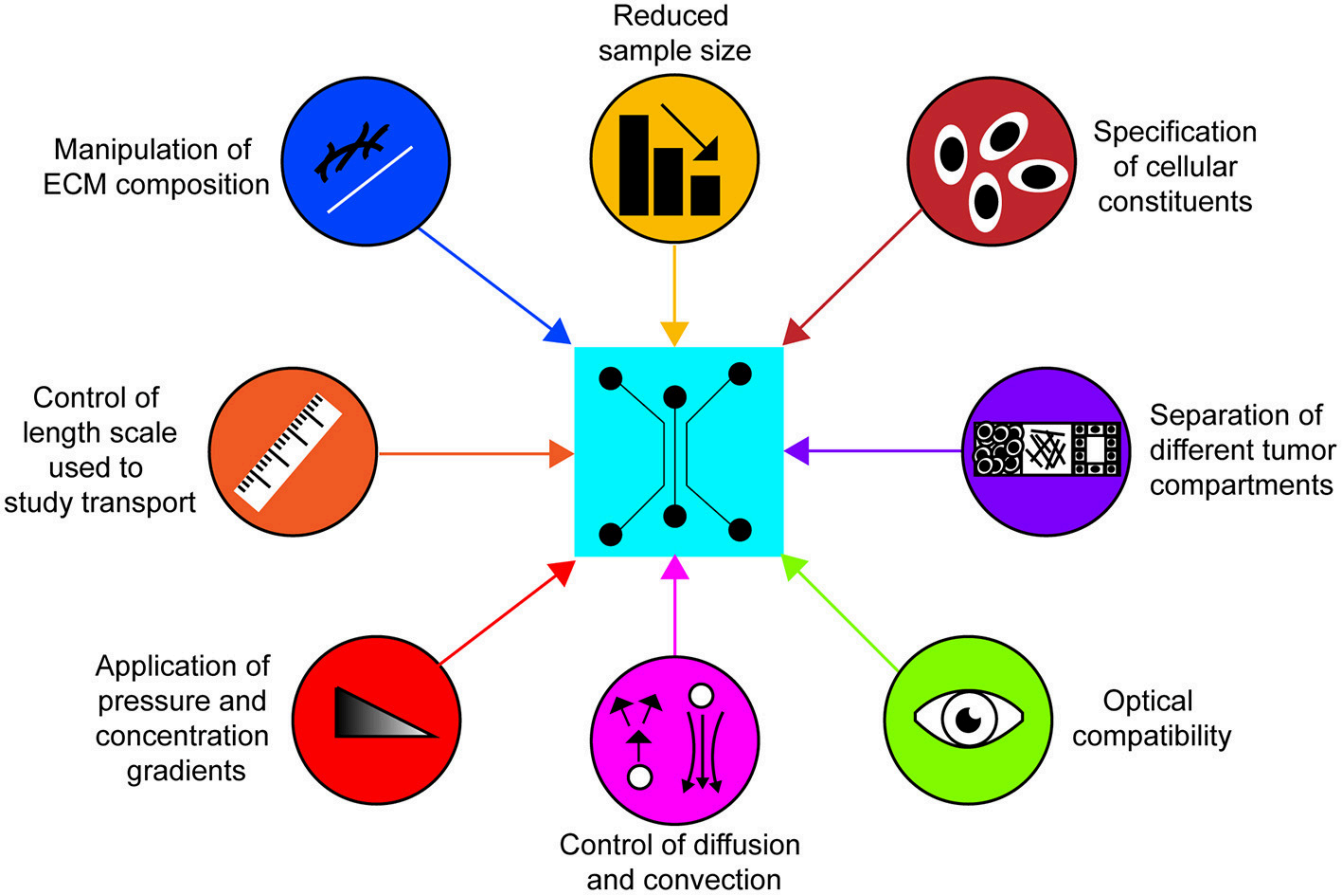
Characteristics of 3-D microfluidic platforms for studying transport in the tumor ECM. Microfluidic platforms possess the capacity to readily integrate these attributes to efficiently quantify transport parameters of the tumor ECM *in vitro* and study how they are affected by different cellular and matrix constituents.

Compared to established techniques, microfluidic devices offer several advantageous characteristics for measuring the transport properties of the ECM (Figure 2). For instance, custom design and fabrication of microfluidic devices offer control over the length scale used to study transport phenomena and the formation of separate compartments that are representative of distinct regions of tumors. In addition, concentration and pressure gradients in microfluidic devices can be specified with relative ease and independently of each other, enabling precise control over convective and diffusive transport. Moreover, microfluidic devices fabricated by rapid prototyping of poly(dimethylsiloxane) (PDMS) permit visualization of the ECM since they are compatible with labeling and imaging techniques such as immunofluorescence, confocal reflectance microscopy, and second harmonic generation imaging. This important quality of PDMS microfluidic devices enables simultaneous interrogation of ECM composition and structure with the measurement of transport parameters. Lastly, microfluidic devices offer control over the spatial positioning of cells and the ECM composition. Of the reconstituted, biologically-derived ECM gels, collagen type I is most widely used due to its prominence as one of the main components of the tumor ECM (Sung et al., 2009; Burkel et al., 2016). However, other ECM gels such as fibrin and Matrigel are commonly used (Ng and Pun, 2008; Moreno-Arotzena et al., 2015). The biological function of the reconstituted matrices can be further modified through the incorporation of other ECM constituents such as GAGs, and PGs that were described in section Physiological Barriers to Transport (Stuart and Panitch, 2008; Yang et al., 2011; Manneschi et al., 2016; Narkhede et al., 2018). The use of cell derived matrices have also been used to provide a more representative tumor ECM in microfluidic devices (Gioiella et al., 2016; Brancato et al., 2018). Collectively these capabilities allow for the quantification of the contribution of each ECM constituent to transport properties. It is noted that the reconstituted ECM gels used for the desired studies can be subjected to material characterization tests (e.g., stiffness measurements, quantification of protein content) to ensure that they match the physiological properties of tumor tissue *in vivo*.

Given the importance of both *D* and *K*′ as measures of transport through ECM, below we provide examples specific to the application of microfluidic platforms in quantifying these two governing parameters.

### Interstitial Diffusivity (*D*)

Microfluidic devices used to measure values of *D* typically feature a compartmentalized 3-D ECM chamber that is flanked by channels or ports that enable controlled application of concentration gradients across the ECM (Zervantonakis et al., 2010; Evans et al., 2014; Wang et al., 2017). Measurement of *D* then involves applying a known concentration of a fluorescent tracer, measuring the concentration profile across the ECM compartment over time, and analyzing the data to obtain a value for *D* (Ghajar et al., 2008; Timp et al., 2008; Zervantonakis et al., 2010). Ghajar et al. demonstrated that increasing fibrin density significantly restricted *D*, which was attributed to increased matrix viscoelasticity (Ghajar et al., 2008). This study also demonstrated that the value for *D* is inversely proportional to the mass of the molecule of interest and presumably the hydrodynamic or Stokes radius (Seo et al., 2014). In a separate study, Albanese et al. incorporated tumor spheroids in a microfluidic device and monitored the accumulation of polystyrene nanoparticles (NPs) in the interstitial spaces as a property of NP size. This study also successfully bridged the gap between *in vitro* and *in vivo* studies by validating the results obtained in the microfluidic device with ones obtained in a murine model (Albanese et al., 2013). Kwak et al. implemented a tumor microenvironment on a chip (T-MOC) model that included capillary, interstitial, and lymphatic compartments with independent control of fluid pressure. This system demonstrated that NP transport is drastically hindered when IFP is higher than the capillary pressure (Kwak et al., 2014). Interestingly, addition of MCF7 breast cancer cells into the interstitial compartment reduced *D* by a factor of 3, presumably due to cell-mediated physical alterations to the ECM structure. Using a microfluidic model that incorporated cell-derived ECM, Gioiella et al. demonstrated that co-culture of MCF7 with normal breast fibroblasts generated an activated stromal tissue that reduced the *D* through the ECM (Gioiella et al., 2016). Taken together, microfluidic platforms have facilitated the quantification of *D* and how it is affected by both properties of the molecule and the ECM setting, with results agreeing with what has been observed *in vivo* (Tomasetti and Breunig, 2018).

### Hydraulic Conductivity (*K*′)

Several studies have leveraged the ability of microfluidic systems in specifying pressure gradients and ECM composition to quantify the hydraulic permeability (*K*) through application of Darcy's Law (Polacheck et al., 2011; Hammer et al., 2017). Typically, *K*′ or *K* measurements in microfluidic devices involve the compartmentalization of ECM material in a microchannel and the application of a known pressure gradient to induce flow. The fluid velocity through the ECM can be approximated with the use of fluorescent tracers. Ng et al. looked at *K* as a measure of matrix integrity, demonstrating that Matrigel can support flow driven cultures for up to 6 h, with collagen matrices (2 mg/mL) also supporting such conditions (Ng and Pun, 2008). However, the reported value for *K* in Matrigel was below physiologically-relevant values. Tran et al. measured *K* through tumor cell aggregates in microfluidic devices at low and high pressures (Tran and Marcos, 2018). This study demonstrated that high intratumoral pressures can result in hydraulic fracturing of tumor aggregates, manifested by increased local *K* values.

*K* has also been estimated in microfluidic devices with the purpose of estimating interstitial flow and shear stress levels local to cells embedded in a 3-D matrix. This approach has shown that *K* can influence interstitial flow and consequently shear stresses on cells, thus altering cell behavior (Polacheck et al., 2011; Li R. et al., 2018). Lastly, *K* has also been used as a parameter for stromal cell-mediated ECM remodeling. Using this approach, Hammer et al. demonstrated that hyperactivation of platelet derived growth factor receptor alpha (PDGFRα) in the stroma reduced *K* and enhanced *in vivo* breast tumor growth (Hammer et al., 2017). Furthermore, it was shown that *K* was rescued to control levels by targeting PDGFR-α with the tyrosine kinase inhibitor (TKI) crenolanib, and extracellular hyaluronic acid (HA) deposited by stromal fibroblasts with hyaluronidase (HAdase). Recently, it was shown that pharmacologic inhibition of smoothened (SMO) in human pancreatic cancer fibroblasts with the compound GDC-0449 (or vismodegib) destabilized phosphatase and tensin homolog (PTEN) (Pitarresi et al., 2018). Moreover, gene expression analysis revealed that treatment of these fibroblasts with GDC-0449 increased hyaluronan synthase 3 (HAS3) expression levels while microfluidic analysis demonstrated that GDC-0449 treatment reduced *K*, which was rescued to control levels with application of HAdase. In this study, loss of PTEN in the stroma was also correlated with enhanced pancreatic tumor growth *in vivo* and reduced overall patient survival. Importantly, the functionality of microfluidics enabled precise control and manipulation of a biological target, and analysis of the functional outcomes of target modulation, which led to the discovery of previously unexplained adverse effects of PDGFR-α hyperactivation and loss of PTEN in stromal fibroblasts.

## Conclusion and Future Perspectives

Microfluidic systems have successfully validated ECM transport properties obtained with other *in vitro* or *in vivo* models. However, we believe that there are numerous opportunities to utilize these systems for future novel discoveries. For instance, matrix normalizing strategies in oncology have so far focused on depleting collagen and HA in tumors, alleviating barriers to drug transport (Chauhan et al., 2011; Li X. et al., 2018). However, other ECM components may also have important and previously unknown roles in mediating drug transport, either directly as a physical barrier or indirectly by activating cells of tumors to deposit increased amounts of collagen and HA. Therefore, successfully elucidating these roles by ECM components besides collagen and HA may lead to new therapies for targeting the matrix that can improve drug transport and patient outcomes. We note that the application of microfluidic models has so far focused on describing the transport properties of desmoplastic tumors such as breast and pancreatic carcinomas that are rich in collagen and HA (Gioiella et al., 2016; Hammer et al., 2017; Brancato et al., 2018; Pitarresi et al., 2018). However, with increasing information on the different ECM compositions across various tumor types (Naba et al., 2016), measurements of the interstitial ECM transport properties can also be applied to non-desmoplastic tumors such as brain cancer to provide novel insights. Given the ability to systematically manipulate both ECM and cellular constituents, microfluidic systems are intrinsically modular, highly versatile, and therefore especially conducive for these types of studies.

Another prospective application for measuring *D* and *K*′ within microfluidic devices can involve rapid screening to predict functional outcomes based on different genetic profiles of tumor or tumor-associated cells. For example, we previously demonstrated that hyperactivation of PDGFR-α in mammary fibroblasts and loss of PTEN in pancreatic fibroblasts resulted in increased deposition of HA with subsequent decreased *K*′ in the ECM (Hammer et al., 2017; Pitarresi et al., 2018). Moreover, the therapeutic implications of this type of approach can be further extended through the incorporation of cancer patient-derived cells or ECM into the appropriate microfluidic system to predict drug response. While no current assays are ready for routine clinical practice (Shamir and Ewald, 2014), microfluidic systems can potentially provide personalized information on the determinants to drug transport. Thus, looking forward, microfluidic systems can serve as a nexus to bring together engineers, cancer researchers, and oncologists to foment advancements in cancer therapy.

## Author Contributions

All authors listed have made a substantial, direct and intellectual contribution to the work, and approved it for publication.

### Conflict of Interest Statement

The authors declare that the research was conducted in the absence of any commercial or financial relationships that could be construed as a potential conflict of interest.

## Abbreviations

ECs: endothelial cells
ECM: extracellular matrix
FRAP: fluorescent recovery after photobleaching
GAGs: glycosaminoglycans
HAS3: hyaluronan synthase 3
HA: hyaluronic acid
HAdase: hyaluronidase
IFP: interstitial fluid pressure
PDGFR-α: platelet derived growth factor receptor alpha
PDMS: poly(dimethylsiloxane)
PGs: proteoglycans
PTEN: phosphatase and tensin homolog
NPs: nanoparticles
SMO: smoothened
T-MOC: tumor microenvironment on a chip
TKI: tyrosine kinase inhibitor.
